# Supplementary material for: Synaptic plasticity facilitates oscillations in a V1 cortical column model with multiple interneuron types
Source: Front Comput Neurosci. 2025 Apr 30;19:1568143. doi: 10.3389/fncom.2025.1568143 (PMC12075120; doi:10.3389/fncom.2025.1568143)
Supplement: Supplementary file 1 [file Data_Sheet_1.docx]

**Supplementary figures:**


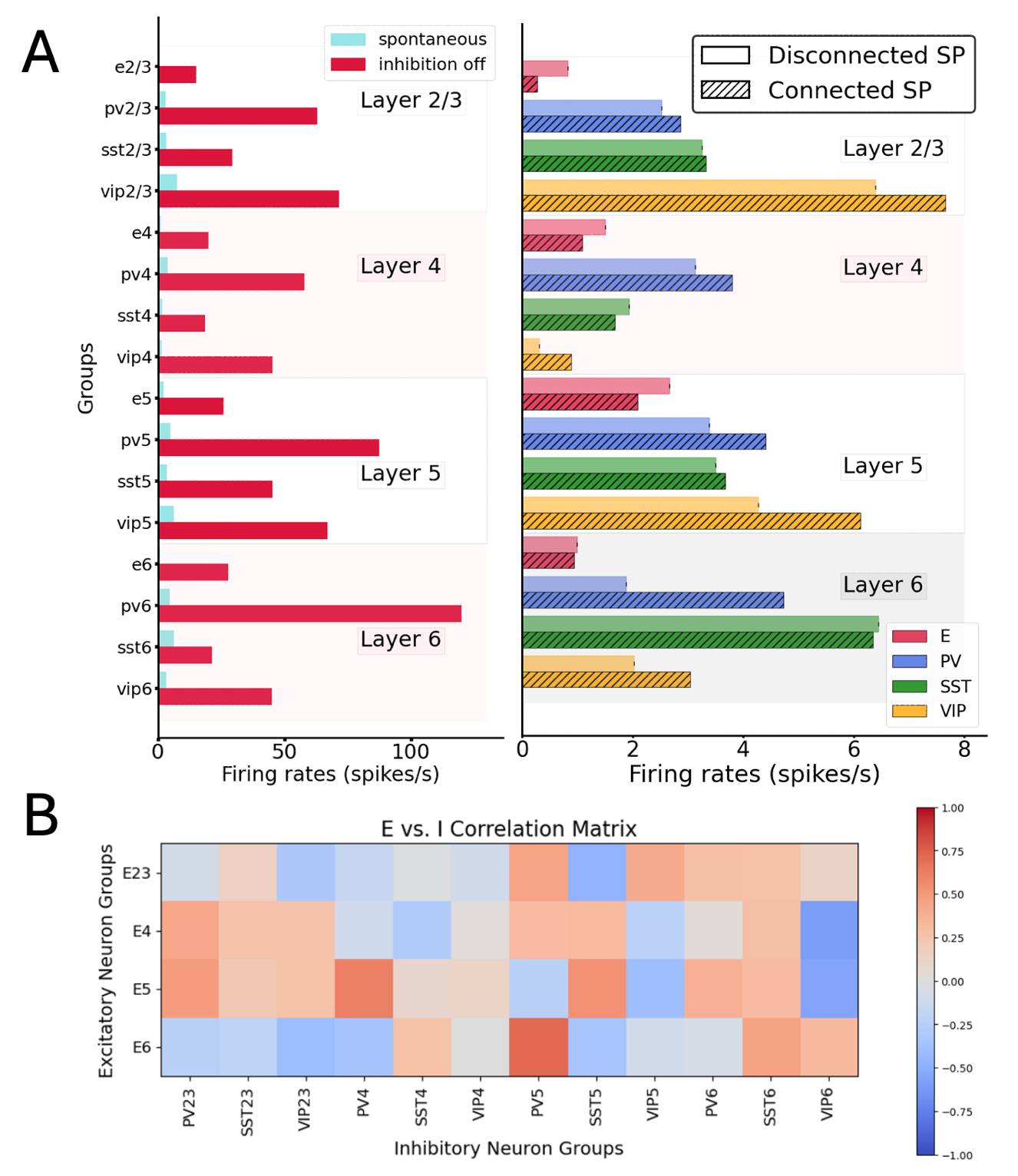


*Figure S1: A, left: Comparison between spontaneous activity in normal conditions (control, blue) vs. the condition of disabling the output from all inhibitory neurons (while maintaining their capacity to spike). All groups substantially elevated their firing rates, particularly those in deep layers. A, right: Comparison between spontaneous activity in the condition of connecting and disconnecting all the recurrent connections inside the column. An overall increase (decrease) in all excitatory (inhibitory) neurons were indicative of an inhibition-dominated network behavior. B: cross-correlation matrix between firing rates of excitatory and inhibitory cell groups across the whole column.*


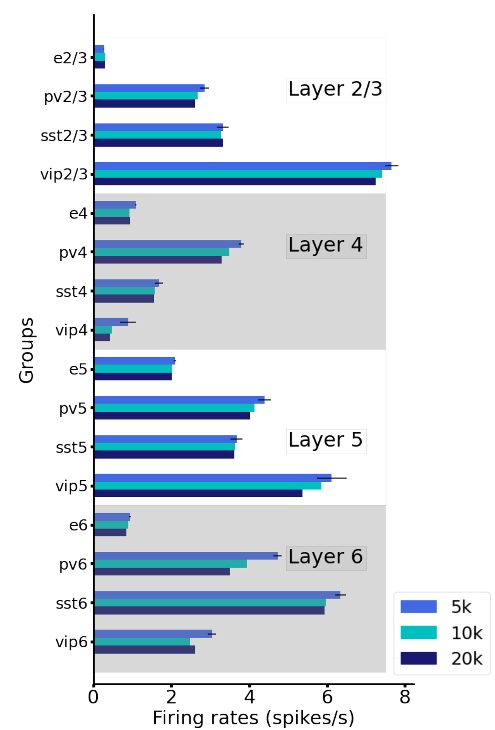


*Figure S2: Mean firing rates of all groups for networks of different sizes. We show that, with proper scaling of the weights (see Methods), results for 5000 neurons, 10000 neurons and 20000 neurons lead to the very similar results.*


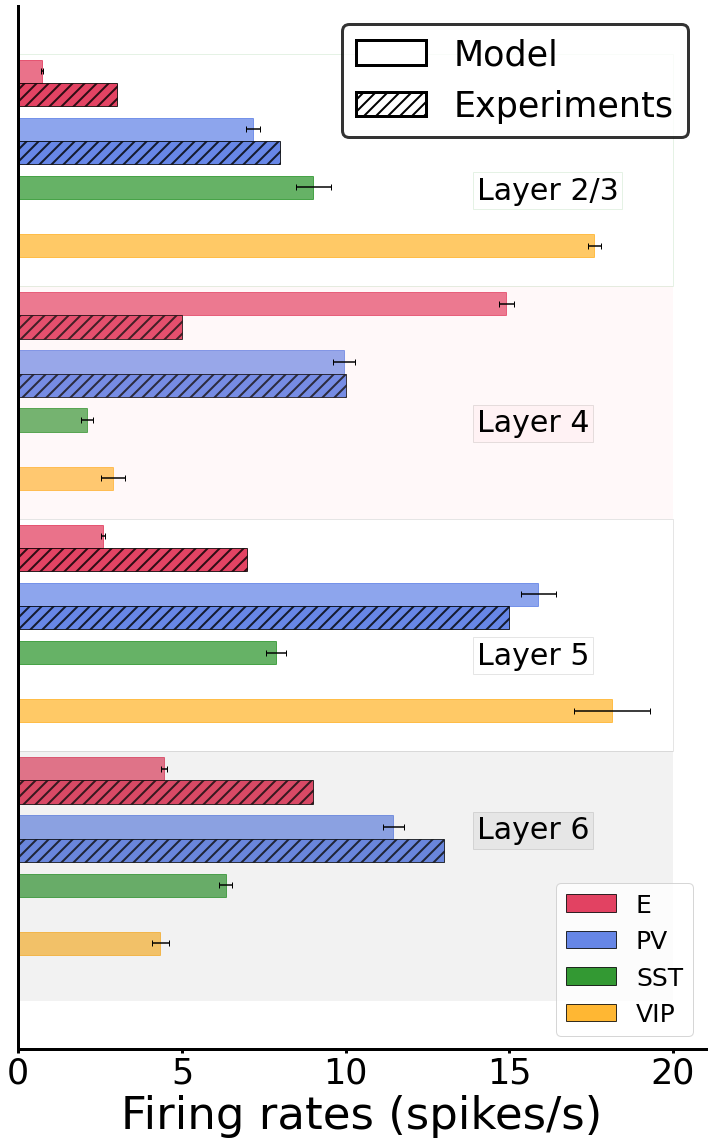


*Figure. S3: Mean firing rates after stimulating layer 4 excitatory neurons (dashed bars). The error bar for the model are computed as standard deviation over 10 different simulations. Error bars for experimental data are not depicted because of its unrealistically significant variations. Boxplots of experimental firing rates can be found in Billeh et al.^22^, in their figure 3F.*

*
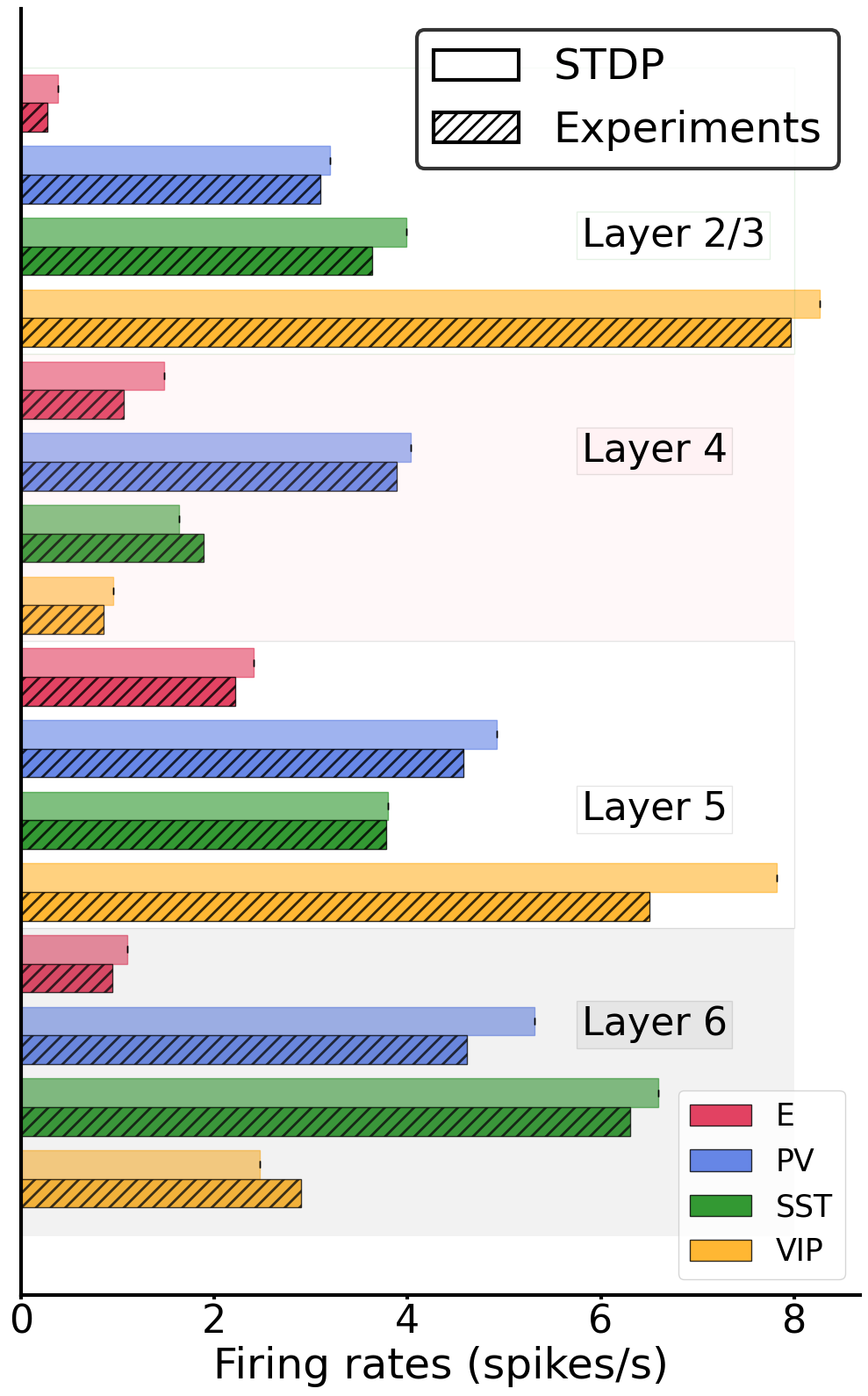

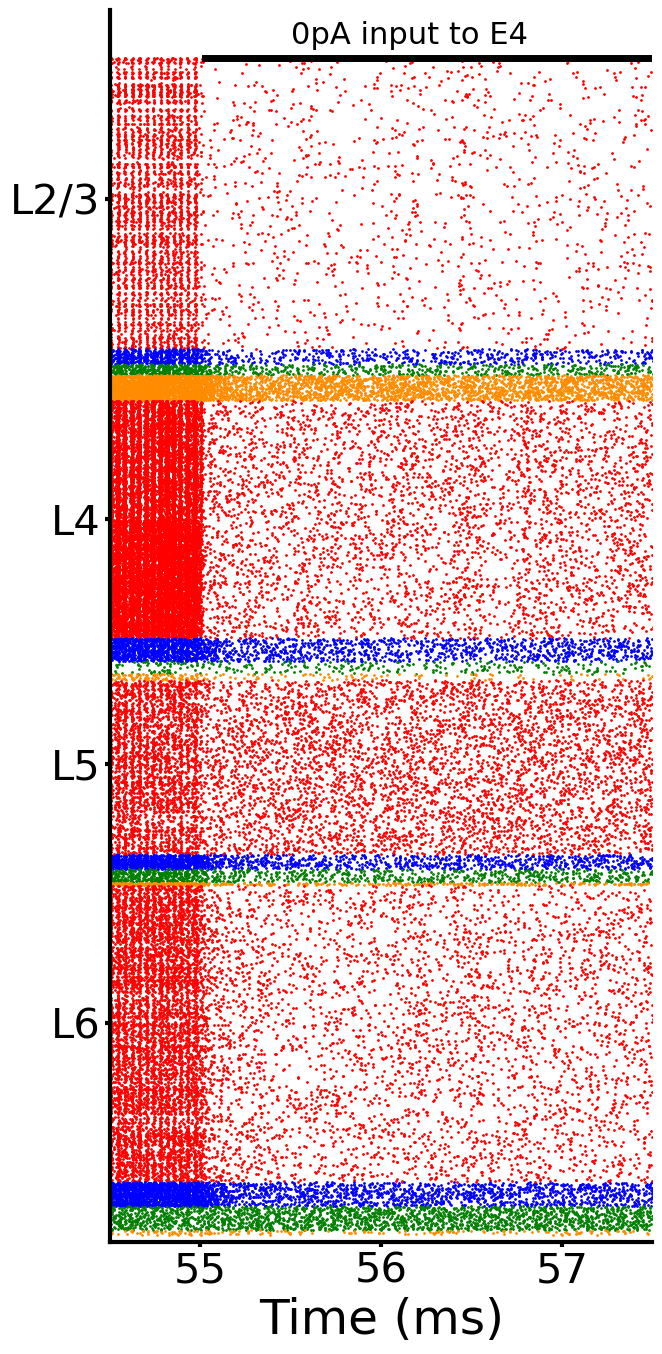
*

*Figure S4:* Spontaneous activity after STDP-induced column. Left: raster plot of spontaneous spiking activity simulated for 1500 ms after plasticity offset (55 s). Right: Mean firing rates for each model population versus experiment.


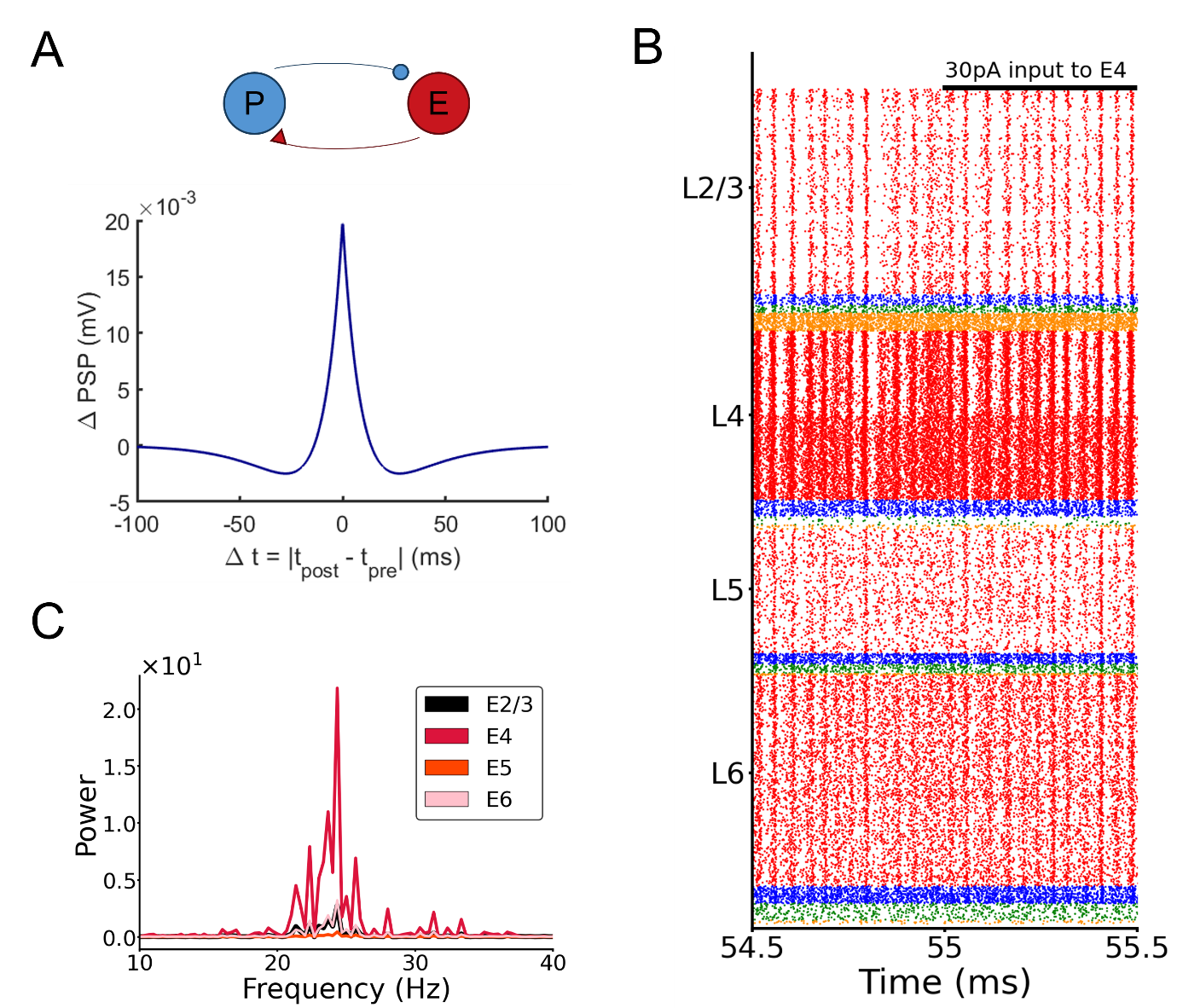


*Figure S5:* Emergence of gamma oscillations in a model with plastic PV-E weights in addition to the plastic E-E weights. (A) Scheme of the inhibitory STDP rule: the relationship between weight changes and relative timing between pre and postsynaptic spikes follows a symmetric “sunken Mexican hat” curve. (B) Raster plot of spike activity in the column with both excitatory and inhibitory plasticity, showing the emergence of oscillations. A continuous input of 30 pA is given to half of the pyramidal cells in layer 4. All excitatory-to-excitatory, as well as all PV-to-excitatory connections in the entire column evolve according to their corresponding STDP rule. (C) Power spectrum of the firing rates of pyramidal cells for different layers, showing a broadening of the curves and a decrease of peak power compared to the results with only excitatory plasticity (Fig. 4C).


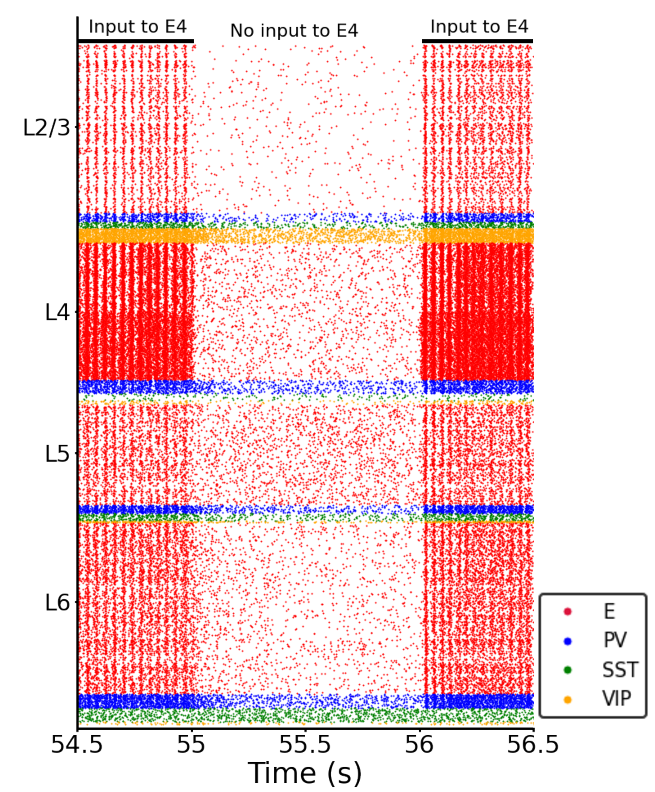


*Figure S6: Raster plot showing the relations between input to layer 4 and oscillations. STDP plasticity was present from the start of the simulation until 55 s. When the input was switched off at t=55 s, the oscillations (frequency: 26 Hz) disappeared; vice versa for switching the input back on. All hyperparameters of this simulations are listed in Tables S3-S10.*


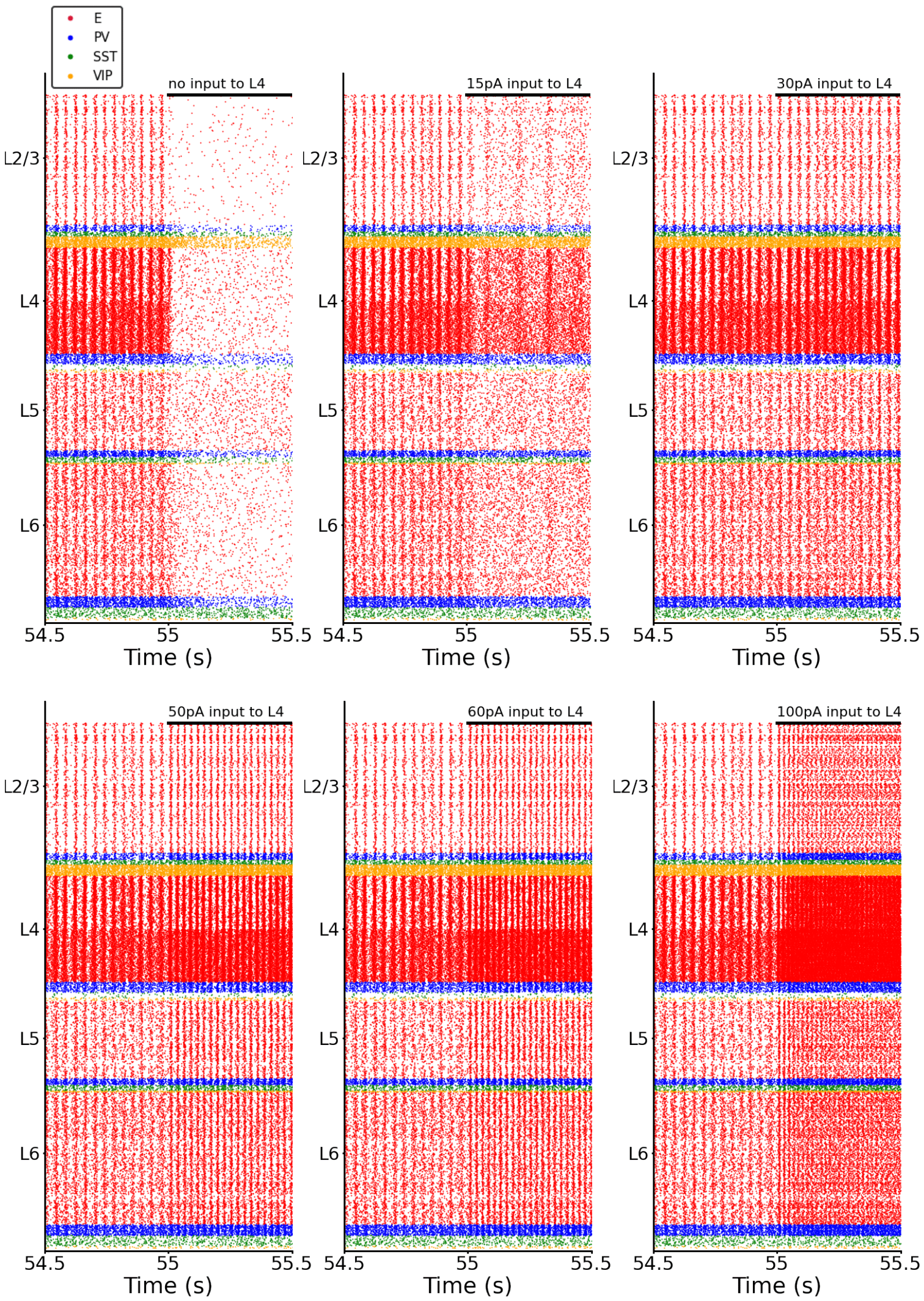

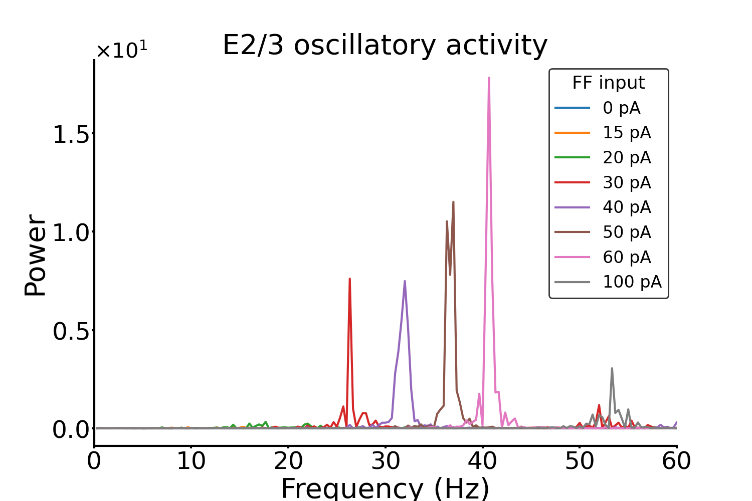


*Figure S7: Modulation of oscillations by feedforward input strength. Top: Raster plots of a subset of the conditions shown in Fig.5A (varying input strength to excitatory cells in layer 4: no input, 15 pA, 30 pA, 50 pA, 60 pA, 100 pA) . Applying more input to layer 4 causes faster oscillations. When no input to layer 4 is present the oscillations disappear. Bottom: Power spectrum of the frequency of excitatory Layer 2/3 neuron firing with varying feedforward input to layer 4 (each color trace represents a different amount of feedforward input to layer 4). The peak amplitudes (max power) and the corresponding frequency are also shown in Fig. 5B and 5C.*


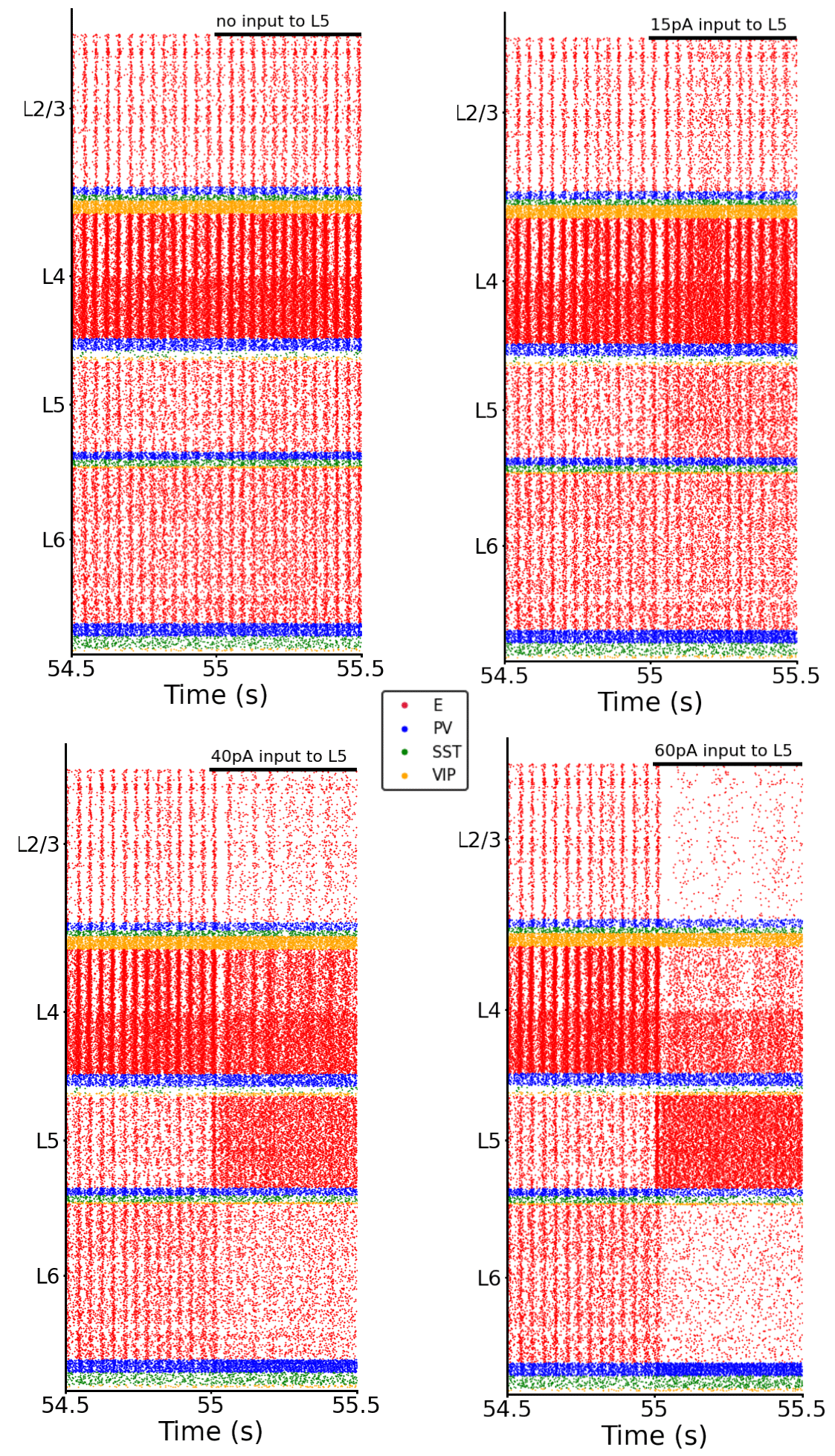

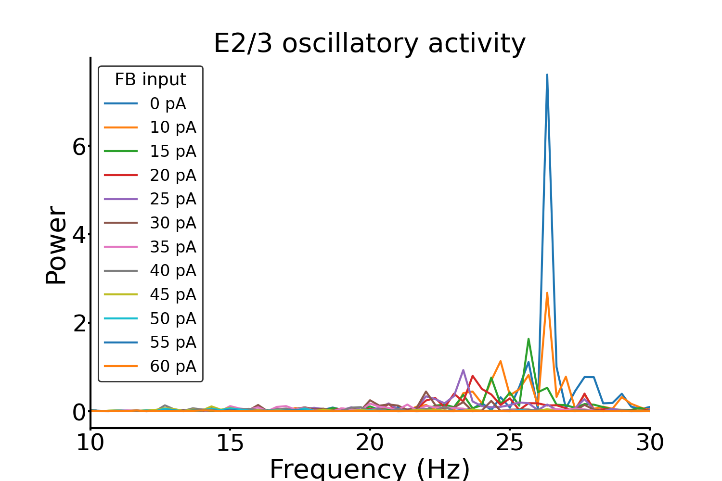


*Figure S8: Modulation of oscillations by feedback (FB) input strength. Top: Raster plots of a subset of the conditions shown in Fig. 5D (varying input strength to excitatory cells in layer 5: no feedback input, 15 pA, 40 pA, 60pA) . The more input is applied to layer 5 (while keeping a constant input of 30 pA to layer 4) the more the oscillations slow down and decrease in power. Bottom: Power spectrum of the frequency of excitatory layer 2/3 neuron firing with varying feedback (FB) input (each color trace represents a different amount of feedback input to layer 5). The peak amplitudes (max power) and the corresponding frequency are also shown in Fig. 5E and 5F.*


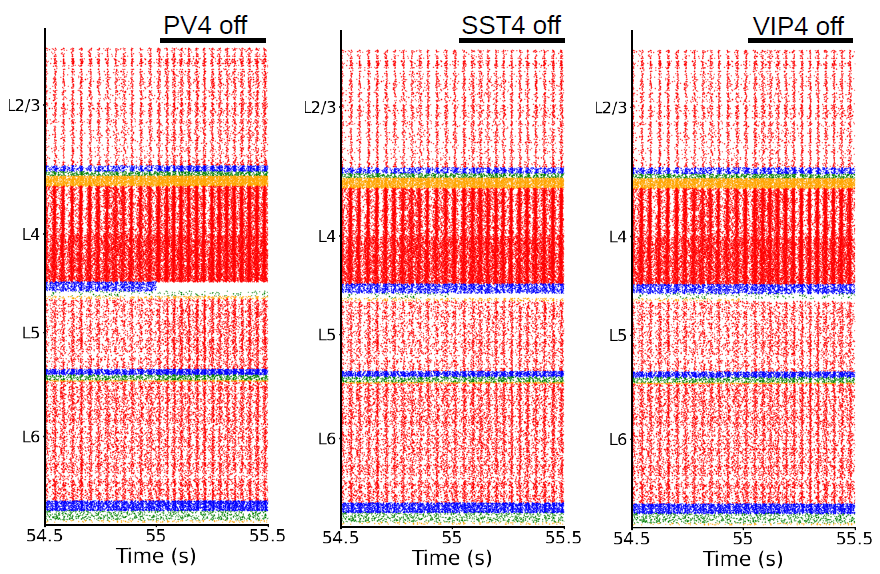


*Figure S9: Raster plots of the whole column model for inactivating different cell groups in layer 4. Each group is inactivated (one at a time) at 55 s to visualize the effects on neural dynamics. From left to right: inactivation of PV neuron in layer 4, SST neurons in layer 4, VIP neurons in layer 4. Removing one group of inhibitory neurons at a time has only a minor effect on the oscillations: a slight increase of the oscillations speed. A bigger effect in increasing the speed of oscillations is however shown in the case of silencing PV cells (leftmost raster plot).*

*
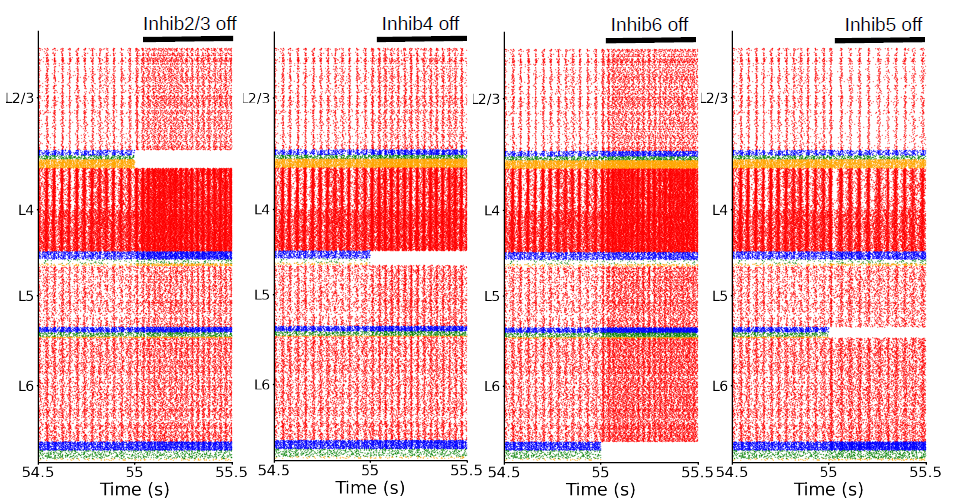
 Figure S10: Raster plots of the whole column model for different inactivation conditions (layer analysis). All inhibitory cells in a layer are inactivated at 55 s, to visualize the effects on neural dynamics. From left to right: inactivation of all inhibitory neurons in layer 2/3, layer 4, layer 6, layer 5. All inhibitory neuron types in each layer contribute to oscillations, removing them one layer at a time shows their effect (increase of oscillation frequency). The inactivation of the inhibitory neurons in layer 2/3 shows the biggest effect. Layer 5 is the only exception: here the oscillations decrease in frequency and power.*


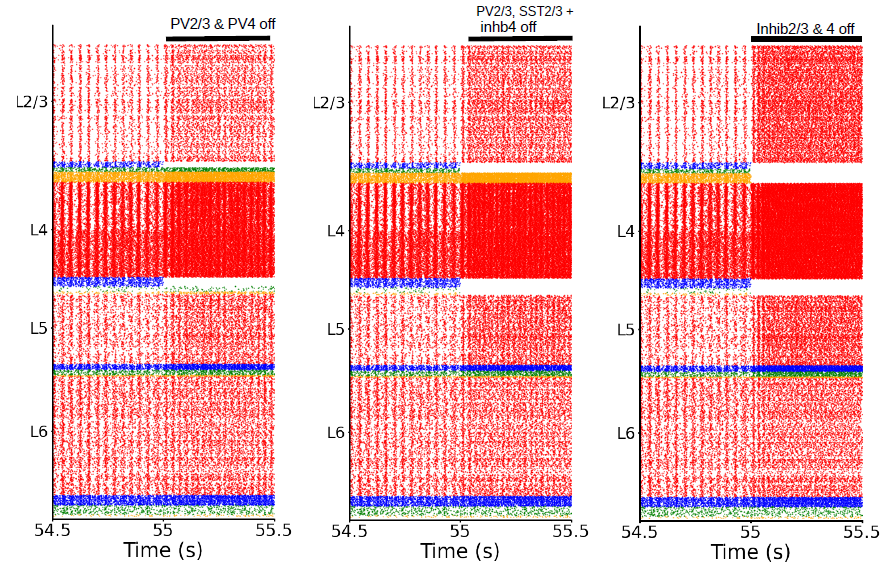


*Figure S11: Raster plots of the whole column model for different inactivation conditions. Each combination of groups is inactivated at 55 s. From left to right: inactivation of PV cells in layer 2/3 and PV cells in layer 4; inactivation of PV and SST cells in layer 2/3; and all inhibitory cells in layer 4, inactivation of all inhibitory neurons in layer 2/3 and 4. Removing more and more groups (from left to right) causes a significant increase in oscillation frequency.*

*
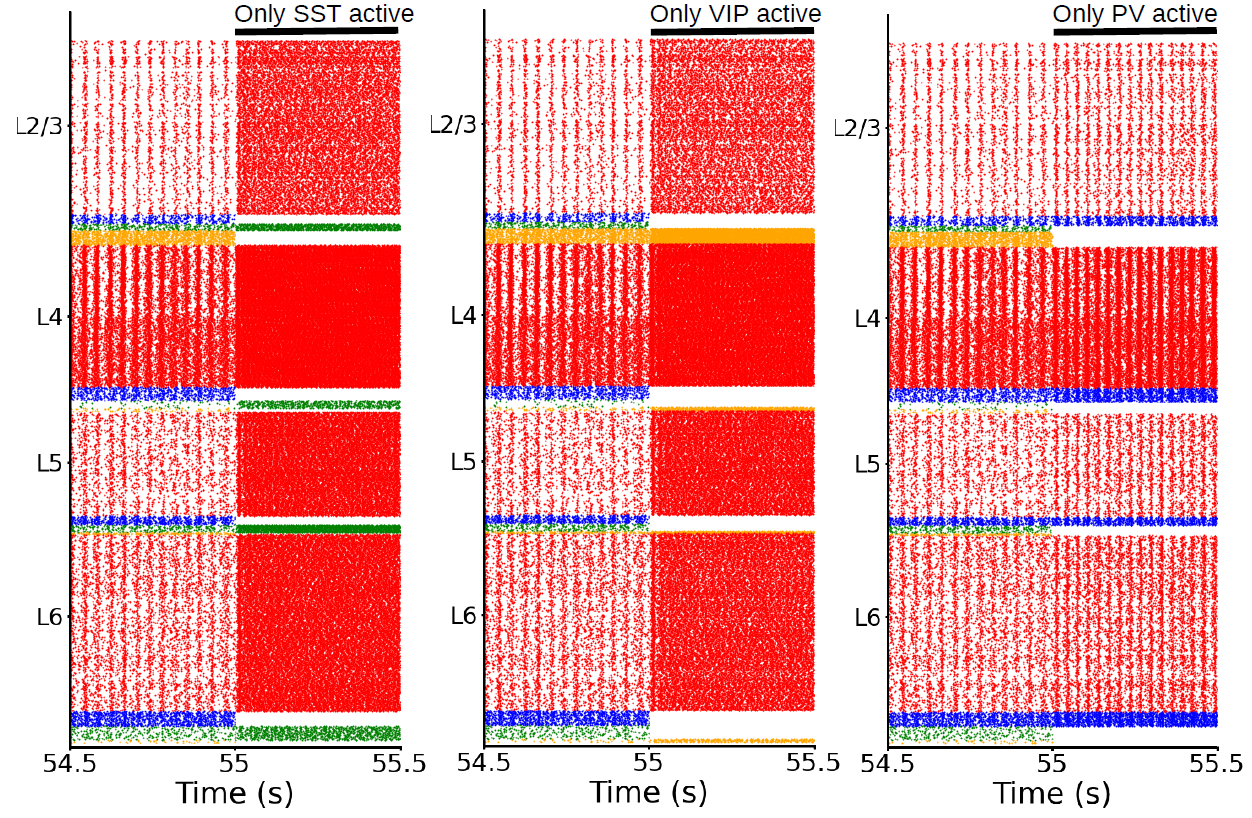
 Figure S12: Raster plots of the whole column model for different inactivation conditions. Each cell type across all layers is inactivated at 55 s. These are experiments where we are leaving active only one type of inhibitory neurons in the entire column (next to pyramidal cells). From left to right: only SST active, only VIP active, only PV active. This shows that – together with pyramidal cells – PV cells alone are able to maintain oscillations around the same frequency (26 Hz). Only a slight increase is visible, the same is not true for SST or VIP. When only them are active the oscillations are drastically affected (first two raster plots).*


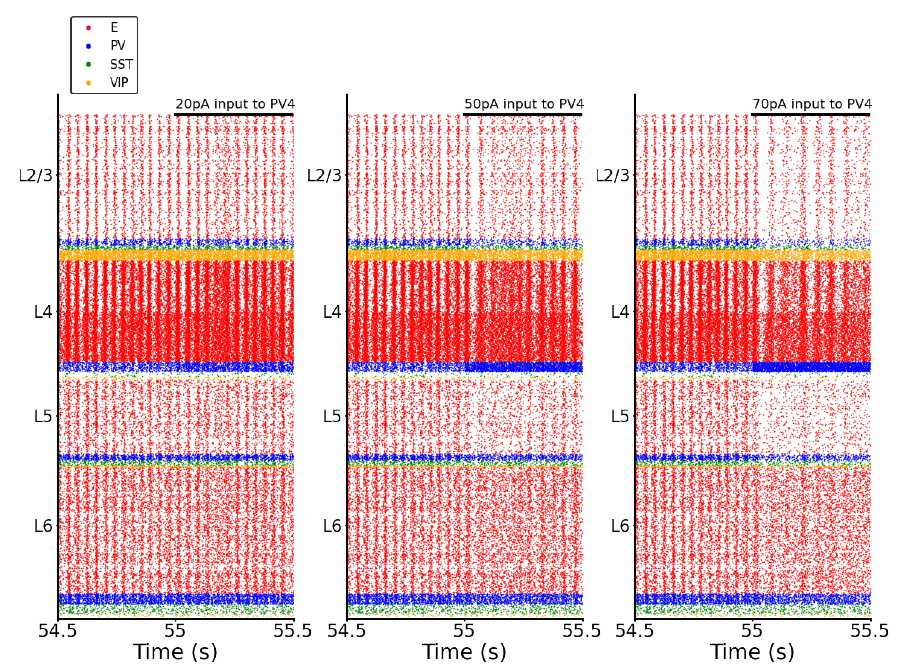


*Figure S13: Raster plots of the whole column model for different input strengths applied to PV neurons in layer 4. The input is given at 55 s. From left to right: 20 pA of input to PV in layer 4, 50 pA of input to PV in layer 4, 70 pA of input to PV in layer 4. Injecting more input into PV cells shows that they are able to modulate the frequency of the oscillations. The more input to layer 4 PV the more the oscillation frequency decreases. In the first two raster plots, a blurring of the oscillations can be observed after 55 seconds. The excitatory neurons are receiving increased inhibition from the PV cells (which are also receiving external input). As a result, the synchrony of their firing, which generates the oscillations, is partially lost, leading to this observable effect.*


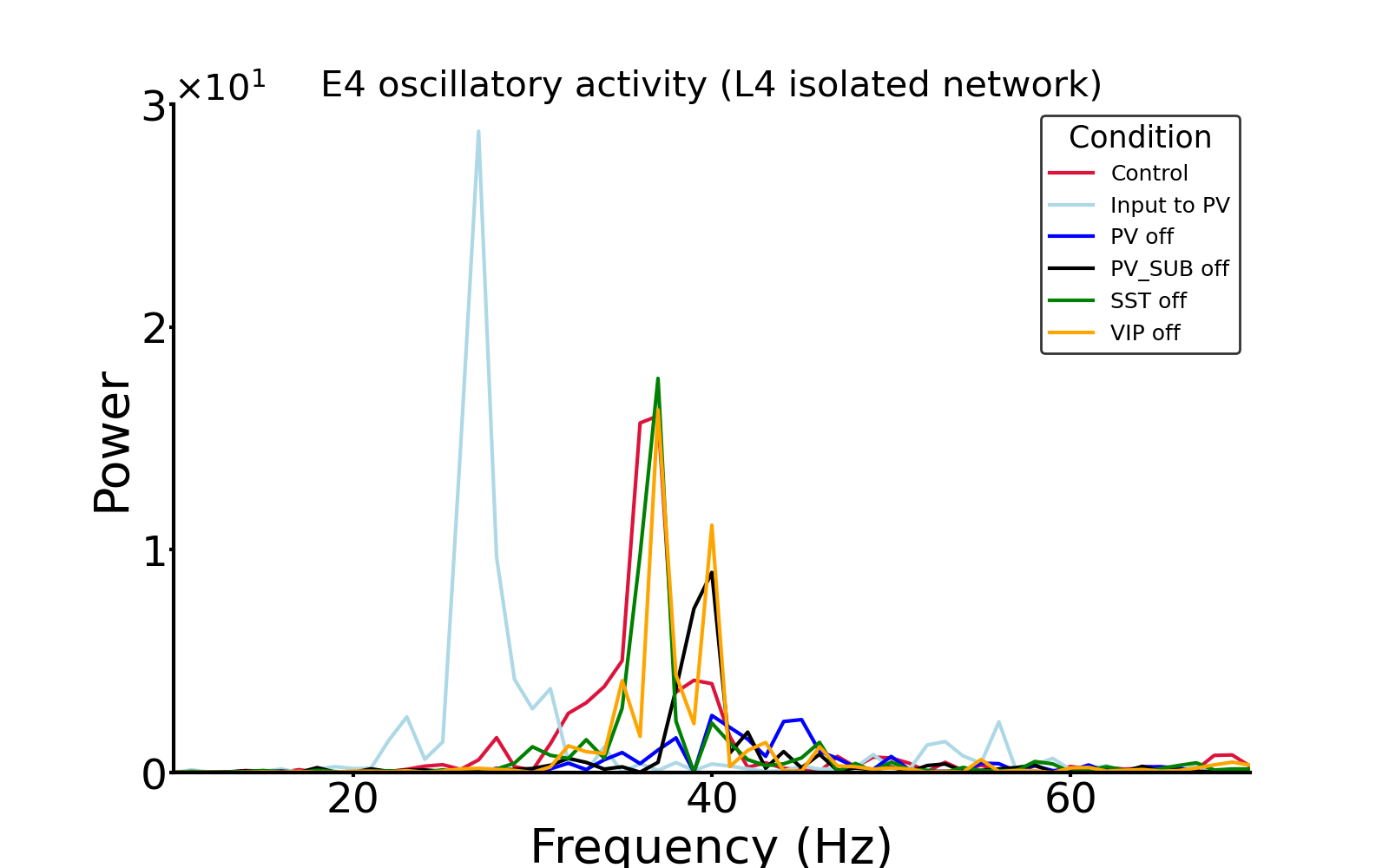


*Figure S14: Power spectrum of the frequency of excitatory neuron firing in layer 4 for different inactivation conditions for the isolated network of layer 4 (schematics in Fig. 6C left). Each color trace represents a different condition, in the legend the name of the group (PV, SST, VIP) represents the inhibited group. Here we can appreciate the shift in the power peak depending on the analyzed condition: when PV cells are inactivated the frequency with max power (peak in the plot) is shifted to the right (blue), indicating an increase in speed of the oscillations. In contrast, when PV cells are stimulated by an external input, they modulate the frequency of the oscillations which shows a significant decrease (light blue trace: Input to PV). This result is consistent with the full-model scenario shown in Fig. S13. A total inhibition of SST cells or VIP cells is not significantly affecting the oscillations (green and yellow trace). The maxima of oscillatory power at 37 Hz for the different conditions were also shown in Fig. 6C (left) as well as the frequency where maximal power was reached (right in Fig. 6C).*

*
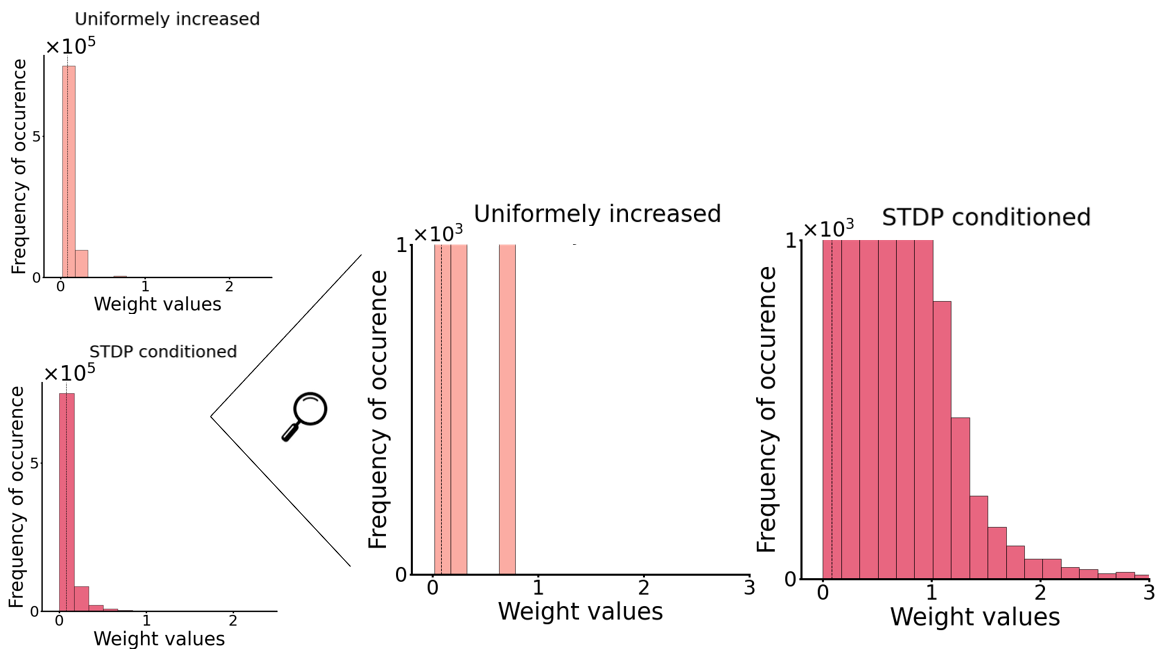
*

*Figure S15: Weighs distribution for the trained network and uniformly increased network (see Fig. 7). Left: distribution of weights for the two networks. Right: zoom in by cutting the threshold on the y-axis at* ${10}^{3}$ *to better visualize the differences between the distributions. Even if the average of all connection strengths of the STDP conditioned and uniformly increased network are the same (mean=0.081) a closer look at the left panel shows that the distributions are different. In the Uniformly increased network, there are few connections (less than 600 out of the total of 800k) that have a high value ( >1.5). Those strong connections are absent in the Uniformly increased network. The reason that the means of the two distributions are so similar, is that in the STDP conditioned network the vast majority of connections are very small (~0), which collectively compensates for the small subset of strong connections. In the uniformly increased network those strong connections are absent, in conjunction with the absence oscillations.*


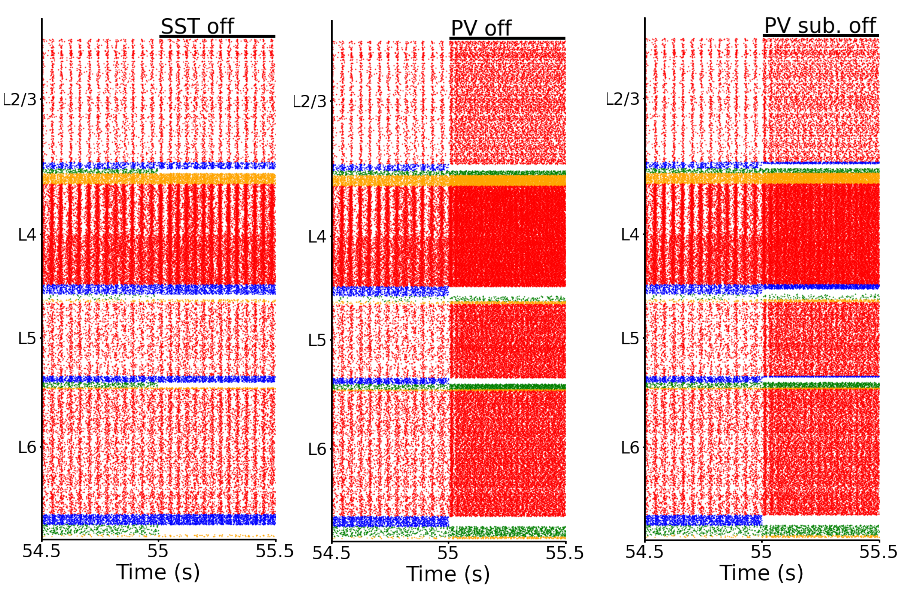


*Figure S16: Raster plots of the entire column model under different inactivation conditions. Each cell type across all layers is inactivated at 55 seconds. The first two raster plots are also shown in 6B. From left to right: the effect of silencing all SST cells, all PV cells, and a subset of PV cells. To test whether the impact of PV cells was not solely due to the higher number of PV cells compared to SST cells, we inactivated a subpopulation of PV cells (PV-sub), equal in size to the number of SST neurons in each layer. The effects on the oscillations, in this case, were significantly stronger than the inactivation of the SST population, thereby proving the stronger role of PV cells. The prominent role of PV cells appeared to be related, not solely to their higher number, but rather to the synaptic connections from PV to pyramidal neurons, which are stronger than the projections from SST to pyramidal neurons.*

*
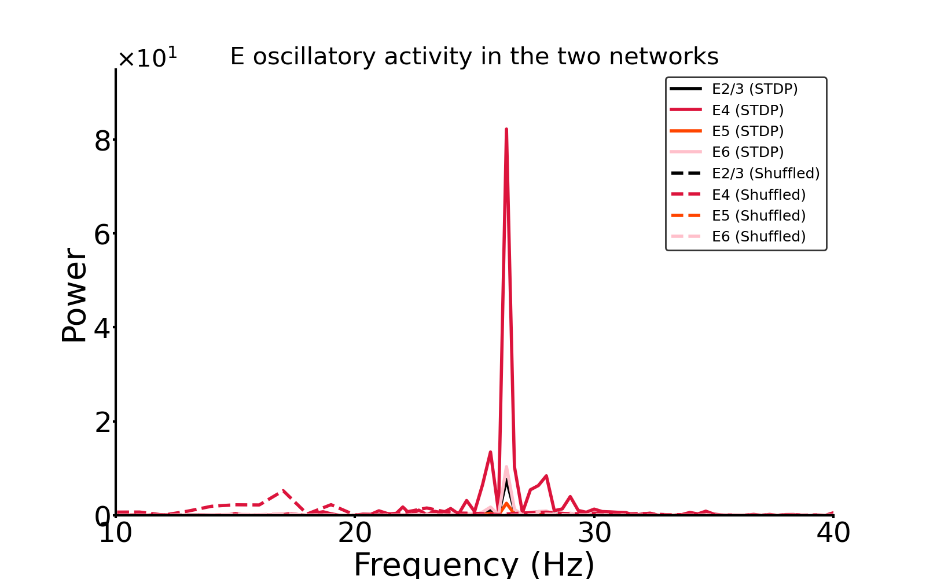

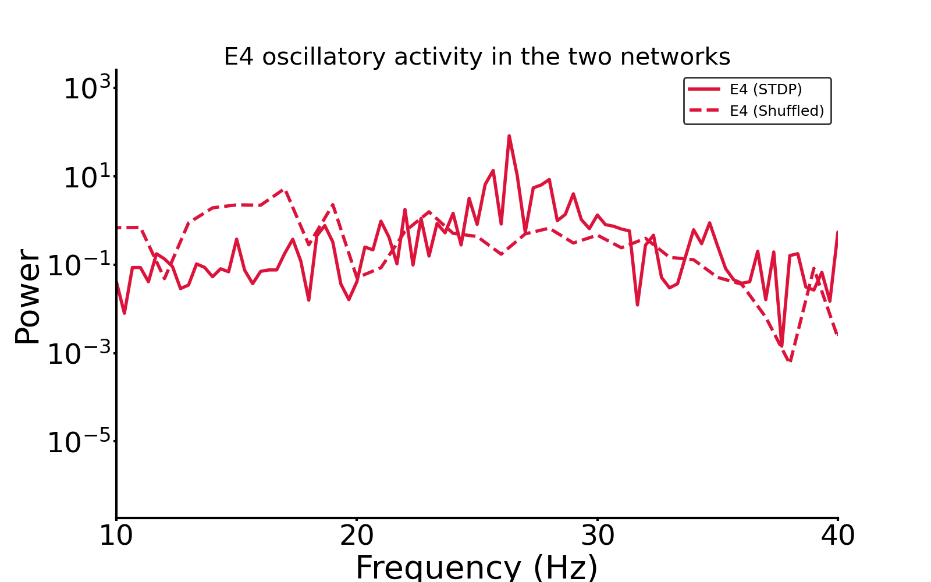

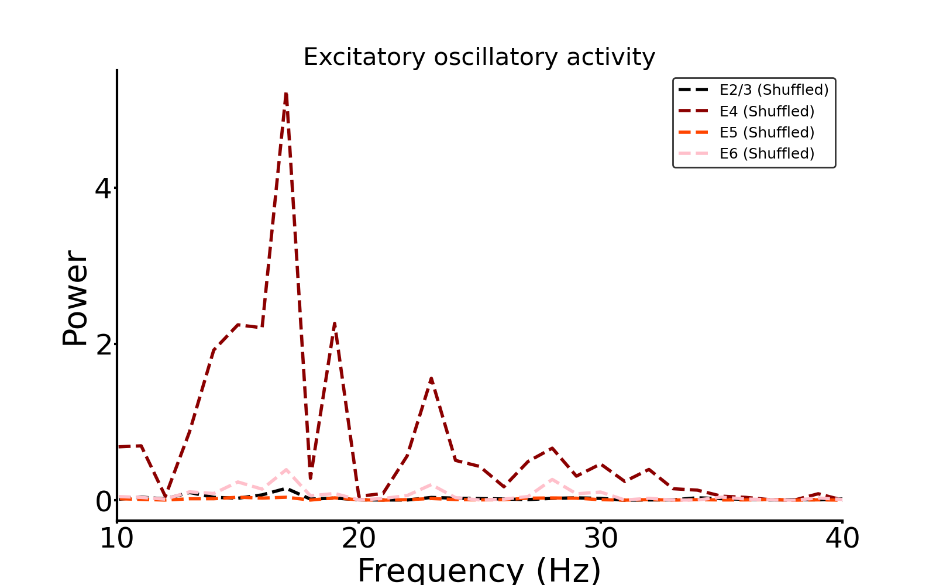
*

*Figure S17: Top: Power spectrum of the frequency of excitatory neuron firing rates in all layers of two distinct networks (STDP conditioned and Shuffled networks). As shown in Figure 7A, in the network with shuffled weights, the oscillatory activity disappears. The peaks at 26 Hz in the Shuffled network are no longer visible. Middle: Power spectrum of the frequency of excitatory neuron firing rates in layer 4 of the two distinct networks (STDP conditioned and Shuffled networks). A logarithmic scale is used to better appreciate the power drop. Bottom: Power spectrum of the frequency of excitatory neuron firing rates in all layers for the Shuffled network. The maximum peak is at 17 Hz; the power at 26 Hz is now irrelevant compared to the STDP conditioned network. In the Shuffled network, the power at 26 Hz drastically drops (e.g., value drops from the scale* ${10}^{0}$*to* ${10}^{-3}$ *for excitatory neurons in Layer 2/3), see also Table 10.*
